# Disruption of model pulmonary surfactant membranes by the cockroach allergen Bla g 1

**DOI:** 10.1016/j.jacig.2025.100544

**Published:** 2025-07-24

**Authors:** Hunter Johnson, Alexander C.Y. Foo

**Affiliations:** Department of Chemistry, St Francis Xavier University, Antigonish, Nova Scotia, Canada

‬‬‬‬To the Editor: ‬‬‬‬‬‬

It is becoming increasing clear that immunologic barrier dysfunction is a key contributor to allergic sensitization and that exposure to airborne barrier-damaging agents likely plays a role in the growing prevalence of allergic airway disease.^1^ Of these agents, the cockroach (*Blattella germanica*) allergen Bla g 1 (full designation Bla g 1.0101 according to the World Health Organization/International Union of Immunological Societies Allergen Nomenclature) is of particular concern. Bla g 1 sensitization is common (17%-45%) and correlated with asthma and other respiratory disorders.^2^ The association between cockroach exposure and/or sensitization and sanitation means that this burden disproportionately affects society’s most vulnerable members, with up to 80% prevalence in some marginalized communities.^2^ The potential of Bla g 1 to disrupt immunologic membranes is thus a key concern. Bla g 1 features a hydrophobic cavity that binds lipids, with fatty acids such as oleic acid (OA) representing its natural ligands.^3^ Recent works by Foo et al show that Bla g 1 can deliver OA directly into the plasma membranes of epithelial cells, perturbing their immunologic function.^3^ However, the applicability of these results to other respiratory barriers remains unknown.

One such barrier is the pulmonary surfactant, the primary component of which is a phospholipid monolayer. The structure and function of this monolayer are sensitive to exogenous lipids, potentially making it susceptible to disruption by Bla g 1.^4^ Here, we show that Bla g 1 perturbs the structure and function of model pulmonary surfactant (MPS) monolayers. Disruption occurs at micromolar concentrations mirroring those used in the study by Foo et al on cell membranes, and requires that the allergen be bound to its natural OA ligands (Fig 1, *A*).^5^ The fluorescent fatty acid analog 11-dansylaminoundacanoic acid (DAUDA) was used to explore this interaction. DAUDA mimics the natural ligands of Bla g 1, binding to its hydrophobic cavity to generate a DAUDA–Bla g 1 complex.^5^ Transferring DAUDA to the MPS lipid phase induces a red shift, differentiating the 2 species. Exposing DAUDA–Bla g 1 to MPS yielded only the allergen-bound species, suggesting adsorption of the intact allergen-DAUDA complex onto the surfactant surface (Fig 1, *B*). This runs counter to its established mode of action against epithelial cells in which OA, but not its allergen counterpart, is incorporated into the membrane (Fig 1, *C*).^3^^,^^5^ Further insight was gained by incorporating DAUDA into the MPS monolayer. Adsorption of Bla g 1 did not displace DAUDA from the surface (Fig 1, *D*), although Apo–Bla g 1 resulted in the formation of the DAUDA-allergen complex. From these results, we propose a potential model in which the intact Bla g 1-OA complex is adsorbed onto the MPS, reducing the available surface area. This increases the packing density of the MPS lipids (Fig 1, *E*). Sequestration of MPS lipids into the unoccupied cavity of the Apo allergen relieves compression, reducing surface activity relative to its OA-bound counterpart.

**Fig 1:**
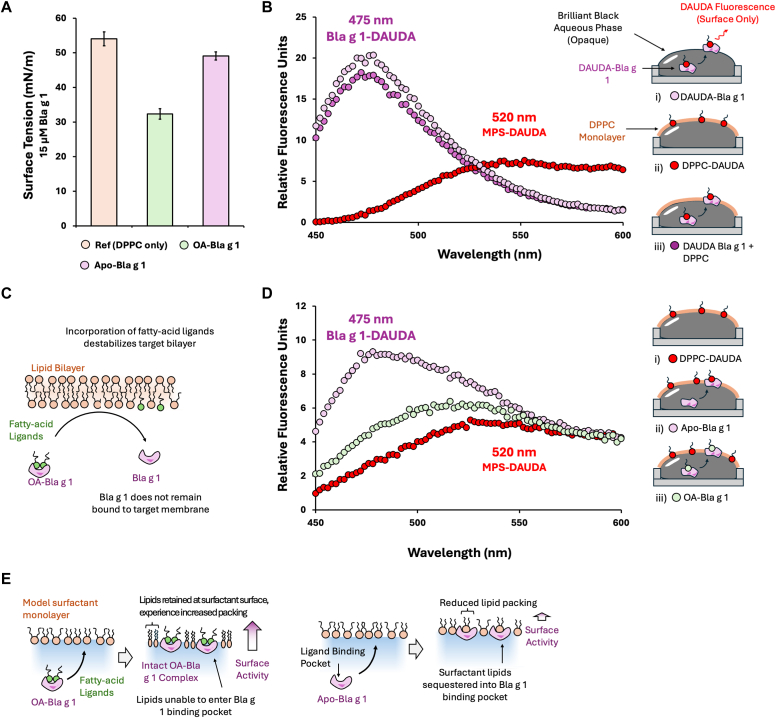
**A,** Impact of Bla g 1 on MPS surface tension. MPS films were created by depositing 3.5 μg of dipalmitoylphosphatidylcholine (DPPC) in methanol atop a 500-μL aqueous phase containing the desired analyte. **B,** Monitoring of Bla g 1 ligand delivery using DAUDA. The aqueous phase contains 0.05% Brilliant Black dye, which blocks all fluorescent signals not originating from the surfactant surface. DAUDA–Bla g 1 and a mixed DPPC-DAUDA monolayer represent the protein-bound (i) and MPS-bound (ii) forms, respectively.^5^ Incubating DAUDA–Bla g 1 with unlabeled MPS yields only the former, suggesting adsorption of the intact complex (iii). Spectra represent averages from at least 3 trials. **C,** Disruption of cellular membranes by Bla g 1.^3^^,^^5^**D,** Monitoring MPS lipid displacement following allergen adsorption. Neither Apo (ii) nor OA–Bla g 1 (iii) reduced DAUDA fluorescence relative to the DAUDA-DPPC control (i), suggesting retention of surfactant lipids at the surface. **E,** A potential model for Bla g 1–mediated surface activity.

Here, we demonstrate the ability of Bla g 1 to disrupt MPS monolayers. Destabilization is dependent on the allergen’s natural fatty acid ligands but it does not appear to be driven by incorporation of these ligands into the lipid monolayer—representing a marked departure from Bla g 1's established behavior against other biologic membranes. Although additional studies are required to confirm this mechanism, these findings expand the potential role of airborne allergens and their codelivered lipid ligands in disrupting immunologic barriers and contribute to the current discourse concerning the impact of these interactions on allergic airway disease and its associated health disparities.

## ‬‬‬‬‬‬‬‬‬‬‬‬‬‬‬‬‬‬‬‬‬‬‬‬‬‬‬‬‬‬‬‬‬‬‬‬‬‬‬‬‬‬‬‬‬‬‬‬‬‬‬‬Disclosure statement‬‬‬‬‬‬‬‬‬‬

Supported by the 10.13039/501100000038Natural Sciences and Engineering Research Council of Canada (Discovery Grant RGPIN-2023-03474 [to A.C.Y.F.], Discovery Launch Supplement DGECR-2023-00224 [to A.C.Y.F.], and Undergraduate Student Research Award 624562 [to H.J.]) and the Government of Nova Scotia Department of Labour, Skills, and Immigration (St Francis Xavier University Building Opportunities for Learning and Development [BOLD] Program [to H.J.]).

Disclosure of potential conflict of interest: The authors declare that they have no relevant conflicts of interest.
